# Relationships between Sleep Patterns, Health Risk Behaviors, and Health Outcomes among School-Based Population of Adolescents: A Panel Analysis of the Korean Children and Youth Panel Survey

**DOI:** 10.3390/ijerph16132278

**Published:** 2019-06-27

**Authors:** Jinseok Kim, Jin-Won Noh, Ahraemi Kim, Young Dae Kwon

**Affiliations:** 1 Department of Social Welfare, Seoul Women’s University, Seoul 01797, Korea; 2 Department of Healthcare Management, Eulji University, Seongnam 13135, Korea; 3 Global Health Unit, Department of Health Sciences, University Medical Centre Groningen, University of Groningen, Groningen 9713 GZ, The Netherlands; 4 Department of Humanities and Social Medicine, College of Medicine and Catholic Institute for Healthcare Management, The Catholic University of Korea, Seoul 06591, Korea

**Keywords:** sleep patterns, health risk behaviors, health outcomes, adolescents

## Abstract

Sleep patterns among adolescents are related to health outcomes and health risk behaviors. This study aimed to describe sleep patterns of Korean adolescents and to find the association between sleep patterns and health risk behaviors and health outcomes. Using the junior high school students’ panel data (n = 2351, 12–15 years old) from the Korean Children and Youth Panel Survey, this study described the sleep patterns operationalized as rising time, bedtime, and sleep duration both on weekdays and weekends. The relationships of sleep patterns with health outcomes and/or health risk behaviors were tested using mixed effect linear regression for continuous health variables and using mixed effect logit regression for binary health variables. Obesity status, the number of chronic symptoms, self-rated health status, smoking, and alcohol consumption were associated with rising time on weekdays after controlling for gender, living area, and housing type. The same set of variables except for the number of chronic symptoms were associated with bedtime during the weekdays. Sleep duration during the weekdays was associated with obesity status, smoking, and alcohol consumption. Similar patterns of association between sleep pattern variables during the weekends and health-related outcome variables were found, but were less obvious than those for weekdays. Significant relationships between sleep patterns and various health-related variables were found among adolescents in Korea. The results from this study indicate that helping adolescents change their sleeping times as necessary to ensure adequate sleep should be considered important in diminishing health risk behaviors and promoting positive health outcomes.

## 1. Introduction

Sleep plays a vital role in good health and well-being. Appropriate, quality sleep helps to protect both physical and mental health and increases quality of life and safety [1]. Especially, adolescents have a physiological need for more sleep [2], and inadequate sleep quantity and quality are linked to significant problems in several health aspects including obesity [3], self-rated health (SRH) [3], smoking [4], and alcohol consumption [4]. In addition, inadequate sleep causes multiple problems in the school setting, where both suboptimal sleep duration and sleep disturbance are associated with reduced academic functioning, including attentional difficulties and increased absences [5,6,7].

Most adolescents experience dramatic changes in sleep and wake patterns [8], including a decrease in sleep duration [9,10,11], a delay in the timing of sleep, and spontaneous morning arousal [2,12,13,14,15,16,17,18], and an increasingly large discrepancy between weekday and weekend sleep patterns [5,18,19,20]. Those changes in sleep patterns are due to the psychosocial pressures of homework, sports, jobs, social activities, and decreases in parental control over bedtime. These pressures combine to reduce the amount of time adolescents spend on sleeping [13,21]. Korean adolescents experience greater inadequate sleep problems than their peers in the West due to a special emphasis on education and obedience to parental aspirations for excessive study based on strong Confucianism traditions [22].

Sleep patterns in adolescence have been consistently shown to be associated with health outcomes [23,24,25,26]. Sleep deprivation caused by poor sleep patterns has the greatest negative effects on the control of behavior, emotion, and attention [27]. Thus, sleep patterns have relationships with health behaviors, like cigarette use, alcohol use, low participation in physical activity, and suicide contemplation [28]. However, only a few studies have been conducted on the association between sleep patterns and health outcomes [23,28,29] or health behaviors [4,28]. Most of these studies focused on sleep duration and not on various aspects of sleep patterns such as rising time and bedtime. The purpose of this study was to describe the sleep patterns and to examine the relationships between sleep patterns, health risk behaviors, and health outcomes among Korean adolescents using nationally representative longitudinal data that indicate the importance of healthy sleep patterns for reducing health risk behaviors and producing better health outcomes.

## 2. Methods

### 2.1. Data and Subjects

This study utilized data from the Korean Children and Youth Panel Survey (KCYPS), a nationally representative study of Korean children and youths. The KCYPS, aiming to investigate various aspects of children’s and youths’ growth and development, started to collect data from three different panels of first and fourth grade elementary school students and first grade junior high school students in 2010 (n = 7071). This same group of students was followed annually until 2016. The KCYPS employed a multi-stage stratified cluster sampling method with schools as the primary sampling unit. The schools were selected using a probability proportional to size sampling method. This analysis used the junior high school student panel (n = 2351) that included participant developmental stages from first year of junior high school up to one year after high school graduation. The participants’ ages at the first wave ranged from 12 to 15 years old. This study was approved by the Institutional Review Board of the Seoul Women’s University (IRB-2018-46) with a waiver for informed consent because the data were obtained from a public data depository which is freely accessible online at http://archive.nypi.re.kr/modedg/contentsView.do?ucont_id=CTX000029&menu_nix=qZc474Ak.

### 2.2. Variables and Measurement

Three indicators measuring sleep patterns of adolescents were utilized in this analysis. Bedtime and wake-up time were ascertained using the question “What time do you usually go to bed and get up?”, which was asked for weekdays and for weekend days separately. Sleep duration during weekdays and weekend days were calculated using the answers for the bedtime and wake-up time questions.

Several health outcomes and health risk behaviors such as SRH, number of chronic symptoms, obesity, smoking, and alcohol consumption were included in this analysis. SRH was measured using a 4-point Likert scale (1 = “very unhealthy”; 4 = “very healthy”). Respondents were asked about any chronic symptoms of asthma, rhinitis, atopic dermatitis, heart disease, diabetes, or other chronic disease experiences during the last year. The number of chronic symptoms experienced during the last year was counted and used in the analysis. Obesity status was measured by calculating the body mass index (BMI) using respondent self-reported height and weight. Finally, health risk behaviors, such as smoking and alcohol consumption during the last year, were measured using respondent self-reports of such behaviors. Gender, regional area, and type of housing were included as control variables. Gender was measured using a dichotomous variable (male = 1, female = 0) and area was also dummy-coded (urban = 1, non-urban = 0). Type of housing had three dummy-coded categories (1 = house, 2 = apartment, 3 = others).

### 2.3. Statistical Analysis

In order to provide sample characteristics, a set of descriptive analyses were conducted. The relationships of sleep patterns with health outcomes and/or health risk behaviors were tested using mixed effect linear regression for continuous health variables and using mixed effect logit regression for binary health variables. The mixed effect regression models were considered appropriate for analysis of panel data structure such as the KCYPS [30]. Stata 15 (StataCorp LP, College Station, TX, USA) was used to manipulate the data and calculate the model parameters.

## 3. Results

The characteristics of the sample are presented in Table 1. Half of the participants in the sample were female. A majority of participants (85.7%) lived in urban areas. In terms of housing types, 59.1% lived in apartments and 20.6% lived in houses.

The proportion of obese adolescents increased from 8.4% in Wave 2 to 16.5% in Wave 6. Female adolescents were less likely to be obese than male adolescents across the study period. Smoking and alcohol consumption behaviors among the adolescents increased over time. In Wave 2, 6.2% and 4.7% of the respondents were smoking and drinking alcohol, respectively, while 11.2% and 27.3% were doing the same in Wave 6, respectively. Again, females were less likely to smoke or drink alcohol than male counterparts throughout the study period. The number of chronic symptoms was asked only in Wave 1 and 4. In both the waves, the adolescents reported less than one chronic symptom with higher number in Wave 1 (mean 0.64, SD 0.78) than in Wave 4 (mean 0.59, SD 0.83). SRH score did not show a consistent pattern of change over time, but male adolescents seemed to report higher scores than female respondents.

Table 1 also presented the overall patterns of sleep variables over the study period. The respondents reported from 6.12 (Wave 5) to 7.90 h (Wave 1) of sleep duration (weekdays) during the study period. Females appeared to sleep less than male counterparts throughout the study period. Overall, the respondents spend less amount of time on sleeping as they got older. Respondents’ bedtime was getting later as they grew up, but rising time did not change as much as the bedtime did (Table 1).

Table 2 summarizes the results of the mixed effects regression analyses of health outcomes and health risk behaviors with sleep duration, bedtime, and wake-up time as predictors after controlling for gender, regional area, and type of housing. Results showed that the number of chronic symptoms was negatively associated with wake-up time on weekdays: The earlier adolescents woke up in the morning during weekdays, the less chronic symptoms were experienced during the last year. The analyses also showed that adolescents going to bed earlier on both weekdays and weekends tended to report higher SRH scores. Additionally, early-rising adolescents tended to report higher SRH scores. Obesity risk was negatively associated with sleep duration both on weekdays and weekends and positively with bedtime and wake-up times both on weekdays and weekends. Bedtime and wake-up times during both weekdays and weekends were positively associated with adolescents’ likelihood of reporting smoking and alcohol consumption behaviors during the last year. Also, sleep duration on both weekdays and weekends was negatively associated with the likelihood of adolescents’ self-reported alcohol consumption (Table 2).

## 4. Discussion

To our knowledge, this is the first large-sample, nationally representative longitudinal study of adolescents to investigate relationships of diverse sleep patterns, health outcomes, and health risk behaviors. Sleep quality including sufficient sleep, earlier wake-up time, and earlier bedtime is associated with better health outcomes and diminished health risk behaviors [23,24,25,26,28,29]. Shorter sleep duration was associated with obesity. These results are consistent with reports that shorter sleep time has contributed to the obesity epidemic [31,32,33,34,35]. Wheaton et al. [35] demonstrated that short sleep duration was linked to unhealthy weight-control behaviors including dieting, fasting, and purging that can lead to being obese. This also can be explained by increased adolescent wake times equating to more opportunities to eat.

Shorter sleep time was positively associated with alcohol consumption [36]. McKnight-Eily et al. [4] found a synergistic effect of short sleep duration and alcohol consumption on school maladjustment, sexual activities, and degraded cognitive abilities. On the other hand, the findings did not show a significant relationship between sleep duration and smoking in contrast to prior studies [4,36,37]. This difference may be due to the low rate of smoking in the sample. In summation, sufficient sleep should be encouraged by enhancing adolescent self-control and parental support. Moreover, children having parents with high health literacy reported significantly longer sleep duration [38]. Thus, parental health literacy may be a key intervention to lengthen the sleep duration in adolescence.

When adolescents go to bed or rise appeared to play a pivotal role in health outcomes and health risk behaviors. Rise time was negatively associated with the number of chronic symptoms. Adolescents going to bed earlier on both weekdays and weekends tended to report higher SRH scores. Earlier bedtime and earlier rise time were linked to higher SRH scores. It is a novel finding that not only total sleep duration, but also bedtime and wake-up time, are related to SRH. Prior research primarily only identified that sleep duration was negatively associated with SRH. This new finding implies the importance of sleep patterns including wake-up and bedtime as well as sleep duration. Goh [39] showed that a 10 p.m. bedtime was less likely to be associated with symptoms of depression than earlier or later bedtimes. Kang et al. [40] found that late awakening leads to higher caffeine intake and severe fatigue among Korean male adolescents. Previous research evidence confirmed that bedtime and wake-up times are crucial factors from medical and behavioral health perspectives.

Earlier wake-up and bedtime also decreased the possibility of obesity. Further, later wake-up time was linked to a higher ratio of smoking, and later bedtime was linked to a higher ratio of alcohol consumption. Miller et al. [41] examined alcohol use as a predictor of sleep patterns among college students. Considering the characteristics of adolescents, alcohol consumption as a consequence of late bedtime may have been more suitable. As such, a large body of literature reported that unhealthy sleep patterns were associated with substance use [28,42], and our findings confirmed the previous findings. Helping adolescents go to bed and rise early should be considered important in promoting positive health outcomes and diminishing health risk behaviors.

This study has several limitations. Although the study shed light on the relationship between sleep patterns and health outcomes and health risk behaviors among adolescents, the findings are only representative of adolescents attending school. Adolescents who do not attend school may be inclined to have adverse sleep patterns and more negative health outcomes in less-structured environments. Thus, future research on these adolescents should be conducted. Second, the data relies on self-report leading to a possibility of inaccurate reporting of sleep patterns. One study found that adolescents underestimated their sleep duration by 30 min [43]. A number of studies also used a self-reported survey questionnaire on sleep patterns [28,41,42,44]. Lastly, smoking and alcohol consumption may have been underestimated because of errors in self-report among adolescents [45,46].

This study contributes to understanding the significant associations between sleep patterns including sleep duration, bedtime, and wake-up time to health outcomes and health risk behaviors. Using a longitudinal approach allowed us to produce valid results. Obesity, the number of chronic symptoms, SRH, smoking, and alcohol consumption were negatively affected by late bedtime as well as late wake-up time. Also, inadequate sleep duration influenced obesity and health risk behaviors including smoking and alcohol consumption. Those effects were revealed for both weekdays and weekends, but the effects were stronger for weekdays. The findings from this study indicate that helping adolescents go to bed and rise early and having enough time to sleep should be strongly considered in promoting positive health outcomes and reducing health risk behaviors. Improving parent, teacher and adolescent awareness of the effects of sleep patterns on health is an adequate beginning. Sleep patterns are a core component of medical or behavioral health interventions for decreasing obesity and preventing health risk behaviors.

This study has implications for research in the field of behavioral health. Further research examining sleep patterns and other factors including cognitive abilities, school adjustment, and relationships with family and friends is needed. Also, future research is needed to examine adolescents’ factors that may moderate these associations. If we better understand the relationship between sleep patterns and health-related factors, intervention studies designed to improve health status by promoting sleep quality should be conducted for adolescents.

## 5. Conclusions

Significant relationships between sleep patterns and various health-related variables were found among the school-based population of adolescents in Korea. Investigating the longitudinal trajectory of the effects of sleep patterns on health outcomes and health risk behaviors can be a next step to further understand these relationships. Identification of healthy sleep patterns for adolescents is needed.

## Figures and Tables

**Table 1:** Sample description.

Variable	Category	Wave 1		Wave 2		Wave 3		Wave 4		Wave 5		Wave 6	
		n	%	N	%	n	%	n	%	n	%	n	%
Gender:	Female	1175	50.0										
Area	Urban	2014	85.7										
Housing type	House	482	20.6										
	Apartment	1385	59.1										
	Other	477	20.4										
Obesity	Total			190	8.4	185	8.2	224	10.7	263	12.7	337	16.5
	Female			64	5.7	65	5.9	80	7.8	98	9.7	133	13.2
	Male			126	11.0	120	10.5	144	13.4	165	15.5	204	19.6
Smoking	Total			141	6.2	147	6.5	189	9.0	229	11	231	11.2
	Female			38	3.4	26	2.3	30	2.9	26	2.5	28	2.8
	Male			103	8.9	121	10.6	159	14.8	203	19.0	203	19.5
Alcohol drinking	Total			107	4.7	160	7.1	272	12.9	444	21.2	562	27.3
	Female			54	4.8	61	5.5	77	7.5	131	12.8	161	15.9
	Male			53	4.6	99	8.7	195	18.1	313	29.3	401	38.5
		**Mean**	**SD**	**Mean**	**SD**	**Mean**	**SD**	**Mean**	**SD**	**Mean**	**SD**	**Mean**	**SD**
Number of chronic symptoms	Total	0.64	0.78					0.59	0.83				
	Feale	0.59	0.76					0.57	0.84				
	Male	0.68	0.80					0.61	0.82				
Self-rated health	Total	3.16	0.61	3.20	0.62	3.21	0.63	3.24	0.58	3.13	0.58	3.23	0.59
	Female	3.15	0.57	3.20	0.59	3.20	0.61	3.18	0.55	3.08	0.54	3.18	0.57
	Male	3.17	0.64	3.20	0.65	3.23	0.65	3.29	0.60	3.18	0.62	3.29	0.62
Sleep duration during weekdays (h)	Total	7.90	0.98	7.67	0.96	7.36	1.01	6.31	1.10	6.12	1.12	6.31	
	Female	7.75	0.96	7.48	0.95	7.19	1.02	6.16	1.06	6.01	1.08	6.20	
	Male	8.05	0.98	7.86	0.94	7.52	0.97	6.46	1.11	6.22	1.15	6.43	
Bedtime during weekdays (h)	Total	23.12	0.93	23.34	0.93	23.66	0.99	24.28	1.04	24.57	1.05	24.51	
	Female	23.23	0.94	23.47	0.94	23.77	1.00	24.34	1.03	24.60	1.02	24.53	
	Male	23.01	0.91	23.22	0.91	23.56	0.96	24.22	1.04	24.55	1.08	24.50	
Rise time during weekdays (h)	Total	7.02	0.48	7.01	0.48	7.02	0.51	6.59	0.51	6.69	0.56	6.83	
	Female	6.97	0.44	6.95	0.47	6.96	0.48	6.51	0.50	6.61	0.54	6.73	
	Male	7.07	0.51	7.07	0.48	7.08	0.53	6.68	0.50	6.76	0.57	6.93	
Sleep duration during weekends (h)	Total	9.52	1.57	9.40	1.59	9.14	1.61	8.53	1.64	8.32	1.63	8.25	
	Female	9.74	1.63	9.61	1.62	9.30	1.55	8.69	1.65	8.40	1.64	8.26	
	Male	9.30	1.48	9.20	1.53	8.98	1.65	8.37	1.61	8.25	1.61	8.24	
Bedtime during weekends (h)	Total	23.76	1.17	23.90	1.19	24.35	1.29	24.86	1.28	25.10	1.25	25.17	
	Female	23.90	1.18	24.05	1.17	24.45	1.28	24.84	1.23	25.07	1.17	25.12	
	Male	23.63	1.15	23.75	1.19	24.25	1.29	24.88	1.34	25.12	1.32	25.22	
Rise time during weekends (h)	Total	9.28	1.64	9.30	1.64	9.49	1.65	9.39	1.65	9.42	1.69	9.42	
	Female	9.64	1.65	9.65	1.65	9.75	1.57	9.53	1.63	9.47	1.64	9.38	
	Male	8.92	1.55	8.96	1.55	9.23	1.69	9.26	1.66	9.37	1.74	9.46	

SD, standard deviation.

**Table 2:** Mixed effects regression analyses of health outcomes and health risk behaviors with sleep patterns.

	Weekday			Weekend		
	Sleep duration B (SE)	Bedtime B (SE)	Wake−up time B (SE)	Sleep duration B (SE)	Bedtime B (SE)	Wake−up time B (SE)
Obesity	−0.003 *** (0.001)	0.006 *** (0.001)	0.003 *** (0.001)	−0.002 *** (0.001)	0.004 *** (0.001)	0.001 ** (0.001)
Smoking (1 year)	−0.03 (0.05)	0.84 *** (0.06)	1.00 *** (0.07)	−0.03 (0.04)	0.73 *** (0.05)	0.52 *** (0.04)
Alcohol drinking (1 year)	−0.07 * (0.03)	0.99 *** (0.04)	1.35 *** (0.05)	−0.13 *** (0.02)	0.80 *** (0.03)	0.40 *** (0.02)
Number of chronic symptoms	−0.01 (0.01)	−0.01 (0.01)	−0.03 * (0.01)	0.01 (0.01)	−0.01 (0.01)	−0.00 (0.01)
Self−rated health	0.00 (0.01)	−0.02 ** (0.01)	−0.03 *** (0.01)	0.02 (0.05)	−0.01 * (0.01)	−0.01 (0.05)

Note: results were controlled for gender, area, and type of housing (i.e., house/apartment/and others). * *p* < 0.05, ** *p* < 0.01, *** *p* < 0.001. SE, standard error.
